# Reproductive Health Surveillance in the US-Mexico Border Region: Beyond the Border (and Into the Future)

**Published:** 2008-09-15

**Authors:** Milton Kotelchuck

**Affiliations:** Maternal and Child Health Department, 715 Albany St T5W, Boston University School of Public Health

This issue of *Preventing Chronic Disease* (*PCD*) celebrates the completion of a highly innovative and bold reproductive health surveillance initiative: the Brownsville-Matamoros Sister City Project for Women's Health (BMSCP). This project, sponsored by the Centers for Disease Control and Prevention (CDC) and made possible through binational institutional collaboration, met the challenge of obtaining comparable reproductive health data on both sides of the US-Mexico border to examine the unique reproductive health challenges found in the border region. Heretofore, no such reproductive health data or mechanism to obtain such data existed or was even thought possible. As the articles and editorials in this issue indicate, the BMSCP has been successful.

As a public health professional who has lived and worked in both countries, I address 3 issues: 1) opportunities and benefits from the BMSCP experience that go beyond the border region and could improve the reproductive health of women throughout both countries; 2) lessons learned to enhance the development of cross-national reproductive health data systems and data collection; and 3) the challenge of sustainability and continued implementation, and even expansion, of the BMSCP model in the other border sister cities.

## New Reproductive Health Information

Several benefits of reproductive health information that go beyond the border region have accrued (or could accrue) from the BMSCP model reproductive health data system. First, Latina reproductive health is relatively understudied in the United States. The BMSCP model, if extended throughout the border region, would provide a major new source of critical and unique information about the reproductive health practices, health care use, and health status of women and infants of Mexican heritage, who made up most of the BMSCP respondents. Information on attitudes about breastfeeding or contraceptive use, for example, could provide valuable insights about Mexican communities far from the border. Moreover, reproductive health information from the Mexican side of the border would allow for an examination of the effect of women's Mexican heritage without the confounding issues of immigration or documentation status.

Second, perhaps because BMSCP's principal focus was the common border region, it chose not to focus on migration issues. Many of the women in Matamoros and other border towns are migrants from the interior of Mexico. Meeting the reproductive health needs of highly mobile populations in both Mexico and in the United States is a public health challenge (one program to address the challenge is the Mexican migrant health program Vete Sano, Regresa Sano [Go Healthy and Return Healthy]) (1). The BMSCP database contains hints about the important impact of migration that call out for future analysis.

Third, the BMSCP is a unique data set to study acculturation (from the US side) and cultural heritage/retention (from the Mexican side). Issues of acculturation (positive and negative) are at the heart of much recent maternal and child health (MCH) literature on immigrant populations. The BMSCP data revealed that women of Mexican heritage on the US side of the border already show some behavioral differences from their counterparts on the Mexican side of the border; they are less likely to breastfeed and more likely to drink alcohol. US residency appears to have begun to change some reproductive health practices (2).

The Latina birth outcome paradox is that birth outcomes among Latina women in the United States (especially first-generation immigrants, who often lack health insurance or extensive education) are comparable to outcomes for US white populations and much better than those in the African American community (3,4). These Latina birth outcomes worsen slightly from the first to the second generation of immigrants. Growing MCH literature suggests that acculturation to the American mainstream over generations has negative consequences for reproductive health and that something in Latina culture is healthier for reproduction. Indeed, researchers found evidence that rates of low birth weight were lower in Matamoros than in Brownsville (2). Why this occurs is not well understood; perhaps protective factors such as social support are lost or new negative factors such as increased exposure to violence and fast food are present. By restricting examination of the Latina paradox to US data, we limit our capacity to understand it. The BMSCP data set could provide critical insight and unique data on acculturation topics such as differential cross-border return migration, characteristics of reproductive-age women (and men) who migrate and those who do not, differences in  nutritional habits, changing social cohesion, and participation in various US health programs.

Similarly, from a Mexican national perspective, the retention of cultural heritage can be seen as the inverse of US acculturation. The Mexican government in recent years has taken many steps to extend Mexican citizenship rights beyond its borders (eg, allowing voting and eligibility for government health programs). The reproductive well-being and birth traditions of women of Mexican heritage (in any country) should be of concern to the Mexican public health leadership.

Additionally, the border region may be its own distinct transnational culture, blending features of both countries into a new hybrid distinct from either Mexican or US mainstream culture. Indeed, cultural blending at borders is often how culture is transformed and transmitted in both directions. The extent to which reproductive health behaviors are blended and transformed at the US-Mexico border is unknown. And although border region culture may be distinct, many women of reproductive age move into it and out of it.

Finally, the BMSCP provides a mechanism for comparisons of reproductive health policy between countries. National policies and practices are difficult to assess, but a natural comparison may be right across the US-Mexico border. Information collected uniformly from women of similar cultural heritage on both sides of the border allows for an examination of the effect of national/regional policies. For example, the Mexican government's normative policies expect breastfeeding to commence within 2 hours of birth, with no hospital distribution of formula or other alternatives, which contrasts dramatically with the absence of breastfeeding-friendly policies in the United States. Immediate postbirth breastfeeding rates (82% for Mexico, 63% for the United States) reflect these policy differences (5). On the other hand, the United States has an aggressive prenatal testing policy for human immunodeficiency virus (HIV) that yielded prenatal testing rates  in Brownsville of 95%, whereas the limited (pre-2007) mandated testing policies in Mexico resulted in a 58% rate in Matamoros (6).

## Improved Reproductive Health Data System Methods

The BMSCP's methodologic experiences also provided new insights and an innovative model to enhance other reproductive health data systems and data collection methods. First, BMSCP demonstrates that cross-national data collection can be standardized between countries with different data systems and achieve a high level of reliability and utility. Binational cooperation facilitated equivalent dual-language data collection instruments, common training for data collectors, common data collection supervision, and centralized database management.

Second, BMSCP showed that a sampling design that used large birthing hospitals could accurately capture the maternal reproductive health experience for all births in the border region. The hospital sample captured 92.7% of Matamoros births and 98.2% of Cameron County births with minimal bias between area registered births and study participant births. Hospitals, especially large hospitals, are now the normative sites for births and can be used to construct high-quality representative sampling frames. Moreover, the BMSCP showed that hospitals would allow and facilitate data collection.

Third, BMSCP showed that a questionnaire similar to that used in the US Pregnancy Risk Assessment Monitoring System (PRAMS), a system that samples new mothers via birth certificates and then collects behavioral, medical, and other data from them 2 to 6 months postpartum by mail and telephone, can be systematically and effectively implemented within 1 to 2 days of birth in multiple hospital settings. This timing of BMSCP data collection lessens the "retrospective" duration of most postbirth data collection instruments, shifts the balance of retrospective vs current information collected, and should decrease recall bias, especially for birthing information.

Fourth, no birth certificate linkages were required to implement the BMSCP model. This is an advantage in Mexico, where birth registration systems are still developing, and in the United States, where 2 obstacles were avoided: obtaining permission to access confidential data and methodologic issues associated with subsequent data linkage efforts.

Fifth, on a practical instrumental level, the BMSCP demonstrated the limits of some questions widely used in US-based survey instruments to classify race or ethnicity, for example, or to accurately self-assess height and weight in this population. BMSCP represents a kind of cognitive testing for survey data items among women of Mexican heritage.

Finally, although the BMSCP was conceived as a model of US-Mexico binational border cooperation, the collection of common data may initially be an easier activity than many others for cross-national public health cooperation. It does not require changes in health care system practices to implement. Moreover, it establishes a precedent (and the personal and professional bonds) to sustain other, more activist interventions and cooperative public health efforts in the future.

## Recommendations for Sustainability

The BMSCP was conceived of as a feasibility project, and it proved to be successful and productive. Its real success will be demonstrated, however, only if it is implemented into an ongoing and comprehensive binational surveillance system of border reproductive health (and ultimately used to help improve border reproductive health and health programs). The authors of the BMSCP editorials and articles in this issue of *PCD *clearly all want to sustain the BMSCP model and encourage its development in all 14 pairs of border sister cities. The border health community should also embrace and vigorously demand its adoption. The vision exists; the challenge is how to get there from here.

Replication and sustainability require a deliberative plan. A few practical suggestions:

- Generate political will. Find more advocates in the border health community and beyond.
- Set up a dedicated advocacy committee.
- Prepare a mission statement and goals.
- Be an ongoing visible presence, and keep proposing a binational border reproductive health surveillance system.
- Encourage an immediate effort to replicate the BMSCP model in 1 more pair of border sister cities. This would start the movement from a demonstration project to translational research. It would provide answers to such questions as the following: Can quality be maintained? Can new local leadership develop? How costly is the project to replicate?
- Develop a specific implementation plan, and possibly start with less ambitious implementation goals. Start with a limited number of sister cities, and then, like PRAMS, slowly build up to a full complement of all sister cities. Data collection could be implemented every other or every third year. Change is slow, but periodicity is needed to see trends.
- Consider having a core staff (eg, interviewers and supervisors) that can move from 1 site to another to save money in training. Create full-time positions and develop experienced staff.
- Address the financing issues head on. Cost should not be the principal barrier to further implementation. The BMSCP estimated its cash outlay was $150,000. The real costs are undoubtedly somewhat higher, as it received much in-kind support from CDC, border hospitals, and the Border Health Department, but the cash outlays will still be small. Sister cities could bid on (and fund) their own area reproductive health data collection efforts. Or states could fund all the sister cities on their borders. Mexican states have indicated a willingness to support personnel costs and help with access.
- Use the data collected by BMSCP in professional publications. The articles presented in this journal are also key to sustainability. Publications extend the value of the BMSCP far beyond the border region. These initial articles are an excellent beginning and hint at the opportunities for further data analysis.

I also recommend some revision and augmentation of the content of the current BMSCP questionnaire to better define its data collection niche, to focus more on key reproductive health issues facing the border area, and to increase its appeal to other nonborder constituencies. Specifically:

**Maternal birthing experiences**

One of the unique features of BMSCP is that it collects data (directly from the mother) right after birth. There is no other ongoing source of information on maternal birthing experiences in the Latina community. The 37% rates of cesarean births on both sides of the border suggest the need for this focus.

**Women's health over the life course**

The BMSCP could be a source of information on preconception and interconception health and health care. Life-course models are increasingly used to explain reproductive health disparities. Diabetes, hypertension, and obesity are all chronic diseases prevalent in the Latina community, and these conditions often originate in the reproductive years.

**Migration history**

The BMSCP questionnaire could obtain more or better migration information than other questionnaires, for example, time migrants spend in the border region, the extent of other family migration, attitudes and expectations about migration, nearby family supports, and health and social service needs because of migration.

**Acculturation/cultural identity/border region identity**

As already noted, the BMSCP provides a superb database to study acculturation and cultural identity. It could explore the strength of that identity and help explain the Latina birth outcome paradox.

**Latina reproductive health**

The BMSCP questionnaire could provide a unique opportunity to examine such topics as theories or hypotheses about Latina social cohesion, attitudes about prenatal care, use of over-the-counter and herbal medicines, and changes in eating habits. This data collection instrument should be directly written to explore Latina health.

**MCH policy relevance**

The BMSCP survey should strengthen its policy focus. It should start directly with policy questions, compare known variations in national policy and practices, and then examine subsequent reproductive health and health care associations. Noticeably underdeveloped were efforts to evaluate participation in or the effect of reproductive health programs (such as the Special Supplemental Nutrition Program for Women, Infants, and Children [WIC]).

This issue of *PCD *celebrates the BMSCP demonstration project's success in obtaining and using comparable binational reproductive health data from the border region. The BMSCP, however, should not be only a onetime success story but deserves to be maintained. Ultimately, the project has enhanced our knowledge base to improve the health and well-being of mothers, infants, and families in the border area and throughout the United States and Mexico.
